# Implementing TeamMAPPS: Formative qualitative findings from the dissemination and implementation study of a new evidence-based team science intervention

**DOI:** 10.1017/cts.2025.22

**Published:** 2025-02-10

**Authors:** Stephen Molldrem, Heidi Luft, Jeffrey S. Farroni, Elizabeth J. Lyons, Kevin Wooten

**Affiliations:** 1 Institute for Bioethics and Health Humanities, The University of Texas Medical Branch, Galveston, TX, USA; 2 Institute for Translational Sciences, The University of Texas Medical Branch, Galveston, TX, USA; 3 Department of Nutrition Sciences and Health Behavior, The University of Texas Medical Branch, Galveston, TX, USA; 4 College of Business, Department of Management, University of Houston Clear Lake, Houston, TX, USA

**Keywords:** Team science, science of team science, team trainings, dissemination and implementation science, implementation science, CTSA

## Abstract

**Introduction:**

Team Methods to Advance Processes and Performance in Science (TeamMAPPS) is an evidence-based Team Science competency model and intervention. TeamMAPPS was developed by experts in the Science of Team Science with translational teams in mind. TeamMAPPS focuses on three core teamwork competencies: (1) psychological safety, (2) awareness and exchange, and (3) self-correction and adaptation. In 2023, the TeamMAPPS framework was operationalized into five online training modules that can be used to train whole teams or individuals, with or without facilitation, in any order. This article reports formative findings from the pre-implementation stage of the TeamMAPPS Dissemination and Implementation (D&I) study.

**Methods:**

We conducted 27 interviews and participant-observation fieldwork with 23 individuals involved in the conceptualization, design, or implementation of TeamMAPPS (four were interviewed twice). All implementers were affiliated with a Clinical and Translational Science Award (CTSA) hub. Data were collected during pre-implementation, when modules were being tested and early-stage implementers were being trained. We used D&I theories and frameworks to structure the study, analyze interview data, and recommend implementation strategies.

**Findings:**

“Adoption,” “reach,” and “effectiveness” emerged as key implementation outcomes. TeamMAPPS was perceived to be evidence-based, highly adaptable, and a Team Science intervention offering unique benefits. We draw on participants’ responses and expert recommendations to suggest implementation strategies.

**Conclusions:**

CTSAs and other organizations can use varied strategies to implement TeamMAPPS. The flexibility of the intervention and its rootedness in an evidence-base synthesized by Team Science leaders make TeamMAPPS appealing for CTSAs seeking to enhance their team training offerings.

## Introduction

The fields of Team Science and the Science of Team Science (SciTS) have been central to the development of new models of teamwork for translational science teams [1–3]. SciTS has steadily developed a body of theoretical and applied research [4–9]. Since 2006, through the Clinical and Translational Science Award (CTSA) program, the National Center for Advancing Translational Science (NCATS) has supported the development of SciTS to help improve team performance [1,10].

Other National Institutes of Health (NIH) Institutes and Centers and the National Science Foundation have also invested in the development of Team Science trainings [9]. These investments were made in recognition of teamwork as an essential ingredient for fostering scientific productivity and healthy innovation ecosystems. Developing educational curricula to impart evidence from SciTS to translational teams and researchers has been critical to the field’s impact.

The need for Team Science training has been chronicled [11–14]. Significant advances have been made in developing and deploying training models and methods [15–18]. Efforts have generated Team Science competencies for trainings and program development [3,19–22]. While there is evidence from meta-analyses that general team training is efficacious [23], the evaluation of Team Science training is not fully established, although recent efforts have been evaluated [2]. Dissemination and implementation (D&I) science has helped refine Team Science trainings and interventions [1].

Team Science Cores at CTSAs have been key disseminators and implementers of Team Science resources [8,17,24,25]. This article reports formative findings from a qualitative ethnographic D&I study documenting the early D&I phases of a new evidence-based Team Science training intervention called “TeamMAPPS: Team Methods to Advance Processes and Performance in Science” [26].

TeamMAPPS was created out of a need within CTSAs to improve team functionality to support translational research. It is deployed as a series of online modules with accompanying implementation support materials (e.g., PowerPoint presentations and facilitation handouts) [26]. The need for strong multidisciplinary team functionality is critically important in translational science, because translational teams require investigators from diverse fields across the translational pathway [27]. The core competencies TeamMAPPS is designed to support are (1) Psychological Safety, (2) Awareness and Exchange, and (3) Self-Correction and Adaptation. These emerge from empirical studies about improving teamwork across numerous team types and contexts. TeamMAPPS content, the online modules, and delivery support materials were developed by subject-matter experts in SciTS, adult education, and the CTSA program. The creation and deployment of TeamMAPPS has been spearheaded by the Team Science Core of the University of Texas Medical Branch (UTMB).

This article reports formative findings from the TeamMAPPS D&I study. Its purpose is to better understand how key TeamMAPPS conceptualizers, module designers, and implementers perceived potential barriers and facilitators to implementing TeamMAPPS. Research involved participant-observation fieldwork and in-depth interviews with TeamMAPPS conceptualizers, designers, and implementers in the pre-implementation phase. *Conceptualizers* included individuals who crafted the ideas for TeamMAPPS and/or its evidence-base; they included research faculty with expertise in the science of teams. *Designers* were adult education professionals and technologists who built the online modules and content delivery materials; they had subject-matter expertise in adult education theory and practice and the design of remote and hybrid learning platforms. *Implementers* were people being trained to deliver TeamMAPPS at their institutions; all were affiliated with a CTSA hub, many with Team Science responsibilities.

Data analysis and reporting was guided by Implementation Mapping, the updated Consolidated Framework for Implementation Research (CFIR), and Reach, Effectiveness, Adoption, Implementation, and Maintenance (RE-AIM) frameworks to identify implementation barriers, facilitators, outcomes, and strategies [28–30]. Whereas CFIR focuses on implementation processes, actors, and settings, RE-AIM focuses on implementation outcomes. To select implementation strategies to overcome anticipated barriers, we used the Expert Recommendations for Implementing Change (ERIC) framework [31], CFIR + ERIC Matching Tool [32], our analysis of participants’ responses, and recommendations of the TeamMAPPS project team (including authors of this article).

### Background of the innovation, its development, and implementation

Highly collaborative, multidisciplinary research teams and efficient translation of evidence into healthcare practice are critical [33–36]. At the same time, researchers often do not receive formal training in skills to work on high-functioning collaborative teams [37–39]. TeamMAPPS was designed to help fill this gap. This pre-implementation study aimed to rigorously apply D&I approaches as part of planning for the efficient translation of TeamMAPPS into CTSA hubs and other institutions. The objectives were to (1) define priority D&I outcomes, (2) identify barriers and facilitators to priority outcomes, and (3) identify potential implementation strategies.

The conceptual basis and evidence-base of TeamMAPPS has been described [26]. TeamMAPPS primarily includes five online learning modules: one covering each of the three core competencies, book-ended by introductory and concluding modules. The modules can be completed in any order and a certificate of completion is issued upon finishing all five.

TeamMAPPS is intended to assist CTSAs in developing high-performing teams by providing them with a highly flexible and evidence-based training intervention that allows for varied implementation strategies. These include full-team trainings, asynchronous learning, as part of courses, for Interprofessional Education (IPE) activities, as a Team Science Core offering, as part of mentorship, and other approaches.

### Implementation theories and frameworks

We utilized Implementation Mapping to orient our study [30]. Implementation Mapping is a D&I approach for developing *implementation strategies* that align with specific priorities and needs of the contexts where an evidence-based intervention is to be delivered. It includes five steps: (1) conducting a needs assessment; (2) identifying actors, outcomes, performance objectives, and determinants; (3) identifying theoretical methods and strategies to facilitate change; (4) producing protocols and materials; and (5) evaluating outcomes [30]. This article reports results from the first three steps, which has facilitated the evidence-informed revision of TeamMAPPS implementation guidance and recommended implementation strategies. As this D&I study continues, we will collaborate with implementing CTSAs to determine which strategies facilitate the greatest impact of TeamMAPPS on target outcomes, with the goal of making additional evidence-informed implementation materials.

To identify key *implementation outcomes*, we used RE-AIM, a widely used D&I model, to deductively identify priority implementation outcomes [40,41]. Understanding the *contextual factors* that shape implementation along with major barriers and facilitators to prioritize RE-AIM outcomes in the settings where TeamMAPPS will be delivered will help maximize impact. Our findings from this pre-implementation study will later be used to develop specific metrics associated with key RE-AIM outcomes. We used the updated CFIR to guide systematic evaluation of constructs in each of its five domains [28]. Table 1 presents key definitions for concepts in the RE-AIM and CFIR models in relation to TeamMAPPS.

**Table 1.** Definitions of key dissemination and implementation framework concepts

Framework	Applied Definition in the TeamMAPPS Implementation Context
**RE-AIM**	
Reach	● Characteristics and number of potential TeamMAPPS participants
Effectiveness	● Positive or negative impacts of TeamMAPPS on teaming behaviors in collaborative research and advances in translational science
Adoption	● Characteristic and number of potential institutions, organizations, and individuals that decide to implement TeamMAPPS
Implementation	● The quality of TeamMAPPS delivery (i.e., implementation fidelity)
Maintenance and Sustainability	● How long TeamMAPPS implementation will be sustained within settings and among participants
**CFIR**	
Innovation	● Characteristics of the components that comprise TeamMAPPS
Outer Setting	● Characteristics of the larger settings in which organizations implementing TeamMAPPS exist (e.g., the CTSA hub network, academic medicine) that may influence TeamMAPPS implementation
Inner Setting	● Characteristics of the internal settings in which TeamMAPPS is implemented
Individuals	● Characteristics and roles of leaders, implementers, and recipients involved in TeamMAPPS
Implementation Process	● Activities and strategies used to deliver TeamMAPPS

CFIR: Consolidated Framework for Implementation Research; CTSA: Clinical and Translational Science Award; RE-AIM: Reach, Effectiveness, Adoption, Implementation, Maintenance and Sustainability; TeamMAPPS: Team Methods to Advance Processes and Performance in Science.

One aim of D&I Science is determining when to prioritize specific implementation outcomes [42,43]. Therefore, this study will not only help identify which outcomes to focus on during implementation but will also contribute to advances in the science of D&I in SciTS. In this article, we focus on “adoption,” “reach,” and “effectiveness” as key outcomes. Designing effective implementation strategies requires a clear understanding of the major potential barriers to achieving priority implementation and effectiveness outcomes [30]. Deductive analyses of pre-implementation study data, using RE-AIM and CFIR, facilitated this planning.

We also used ERIC to *select implementation strategies* that maximize priority implementation outcomes [31]. We used the CFIR-ERIC Matching Tool, which identifies ERIC implementation strategies that are most promising for overcoming known key CFIR-based barriers [32]. We compared participant suggestions with those produced by the CFIR-ERIC Matching Tool and in initial implementation materials. Based on our analysis, we suggest strategies that have the greatest potential to overcome anticipated barriers and achieve priority implementation outcomes.

## Materials and methods

Multiple qualitative approaches were used: (1) participant observation of a two-day online “Train-the-Trainer” with all early implementers in March 2023; (2) ethnographic conversations with TeamMAPPS conceptualizers, module designers, and implementers after the Train-the-Trainer; and (3) in-depth interviews with TeamMAPPS conceptualizers, designers, and implementers. SM led participant-observation at the Train-the-Trainer with support from EL, JF, and UTMB ITS staff in group “debriefs” at key junctures and at the end of each day. SM took fieldnotes and made memos in a virtual document shared with the D&I study team. SM also conducted follow-up fieldwork, including participating in an implementing institution’s “Team Science Day” where he presented about TeamMAPPS and discussed implementation strategies with two implementers and a conceptualizer. SM also had ethnographic follow-up conversations at a mid-2023 Team Science conference with an implementer and conceptualizer and during a key meeting of conceptualizers and designers in mid-2023. SM wrote notes and reflective memos after ethnographic discussions and fed findings back into the D&I study and TeamMAPPS team during regular meetings. Verbal consent was obtained at the start of ethnographic interactions and regularly verbally reaffirmed.

The main source of data for this article are interview transcripts. From February to June 2023, SM conducted 27 in-depth interviews with 23 participants involved in the creation, design, or implementation of TeamMAPPS. Four participants were interviewed twice – before and after their participation in the Train-the-Trainer. Four participants were conceptualizers, four were designers, and 15 were implementers. All implementers were affiliated with a CTSA, many in Team Science roles. Interviews took place over teleconferencing, were audio recorded, and averaged about an hour. SM utilized an in-depth interviewing approach, using a guide organized around D&I concepts and designed to explore participants’ relationship to TeamMAPPS, Team Science, SciTS, and translational science. Interviews used the same basic guide but focused on different issues depending on the participant’s role. Verbally recorded consent was obtained at the start of interviews; demographics were asked at the end. Interview audio was professionally transcribed. The UTMB IRB approved the study (#22–0249). Table 2 reports characteristics of interview participants.

**Table 2.** Participant characteristics

	Number (n = 23)
**Participant type**	
TeamMAPPS Conceptualizer	4
TeamMAPPS Designer	4
TeamMAPPS Implementer	15
**Highest education level**	
Some graduate school	3
One or more non-terminal master’s degrees	4
PhD (not including MD/PhDs)	11
Other terminal doctoral degree (non-PhD, non-MD)	3
MD or MD/PhD^**^**^	2
**Gender Identity**	
Man/Male	9
Woman/Female	14
**Race self-identification**	
White or Caucasian	21
Other race^	2
Sexual Orientation*	
Straight or heterosexual	20
Other sexual orientation^	2
Decade born in*	
1940s or 50s	4
1960s	3
1970s	9
1980s or 90s	6
**Type of area in which they work**	
Urban area	17
Suburban area	6
**Region of the United States**	
Southeast	3
Southwest	13
Midwest	4
Other region	3
**Years worked as a researcher**	
10-15	5
16-25	6
26+	5
Did not identify as a researcher	7
**Class self-identification**	
Middle class	16
Upper class	6
Something else	1
**Country of citizenship**	
United States	23

^ Individual variables collapsed together to protect confidentiality because the number who answered was below 3.

* Total does not add up to 23 because some participants did not report that characteristic.

Participants were highly educated, predominantly white and middle class, and almost equally spilt by gender. The sample was not diverse along lines of race, ethnicity, or sexual orientation.

All study team members are experienced in qualitative research and are members of the UTMB Team Science Core and TeamMAPPS leadership team. The embedded nature of the team makes this D&I study particularly qualitatively robust and attentive to the complexity of implementing Team Science interventions in CTSAs. Utilizing a constructivist worldview, the study team understands its proximity to the intervention and involvement in implementation as a resource and source of insight and reflexivity that guides and strengthens the inquiry [44].

SM, JF, EL, and HL used CFIR and RE-AIM in a deductive thematic analysis of interview transcripts using a consensus coding process [44,45]. The codebook contained three code blocks: (1) the five RE-AIM outcome codes [46], (2) the five updated CFIR domain codes [28]; and (3) the three TeamMAPPS competency codes [26]. We adopted definitions from the frameworks; through discussion during consensus-building, coders identified exemplary types of responses to be captured under each code and refined our application as we developed consensus. The codebook is represented in Table 3.

**Table 3.** Codebook with definitions

Code	Code Block	Definition	Refer to:
Reach	RE-AIM	“The absolute number, proportion, and representativeness of individuals who are willing to participate in a given initiative, intervention, or program.”	RE-AIM, [29,40,46]
Effectiveness	RE-AIM	“The impact of an intervention on important outcomes, including potential negative effects, quality of life, and economic outcomes.”	
Adoption	RE-AIM	“The absolute number, proportion, and representativeness of settings and intervention agents (people who deliver the program) who are willing to initiate a program.”	
Implementation consistency	RE-AIM	“At the setting level, implementation refers to the intervention agents’ fidelity to the various elements of an intervention’s protocol, including consistency of delivery as intended and the time and cost of the intervention. At the individual level, implementation refers to clients’ use of the intervention strategies.”	
Maintenance and sustainability	RE-AIM	“The extent to which a program or policy becomes institutionalized or part of the routine organizational practices and policies. Within the RE-AIM framework, maintenance also applies at the individual level. At the individual level, maintenance has been defined as the long-term effects of a program on outcomes after 6 or more months after the most recent intervention contact.”	
Innovation	CFIR	“The “thing” being implemented”	Constructs in Updated CFIR, [28]
Outer setting	CFIR	“The setting in which the Inner Setting exists”	
Inner Setting	CFIR	“The setting in which the innovation is implemented”	
Individuals	CFIR	“The roles and characteristics of individuals”	
Implementation process	CFIR	“The activities and strategies used to implement the innovation”	
Psychological safety	TeamMAPPS Competency	Use of term or direct response to question about the term	[26]
Awareness and exchange	TeamMAPPS Competency	Use of term or direct response to question about the term	
Adaptation and correction	TeamMAPPS Competency	Use of term or direct response to question about the term	

CFIR: Consolidated Framework for Implementation Research; RE-AIM: Reach, Effectiveness, Adoption, Implementation, Maintenance and Sustainability; TeamMAPPS: Team Methods to Advance Processes and Performance in Science.

SM, JF, EL, and HL met regularly from July 2023 to January 2024 to complete the analysis. Coding was conducted in Atlas.Ti. The team began by discussing CFIR, RE-AIM, and the definition of each code to align on application.

SM, JF, and EL then each coded the same transcript separately, discussing it during several meetings by reviewing the coded transcript together to identify differences and similarities in coding. Discussion about disparate and concordant code application led to greater alignment, with discussions generating consensus. Ambiguities were assisted by HL. After the first transcript was discussed, the process was repeated for three more until there was consistent alignment on code application across analysts and confidence in consensus was achieved.

The remaining transcripts were then divided between SM, JF, and EL and individually coded. The group met weekly to discuss the process and ensure continued consensus. This first round of coding aimed to identify priority RE-AIM outcomes, which were identified as “adoption,” “reach,” and “effectiveness.” Priority RE-AIM outcomes were determined by the frequency at which codes were applied.

**Figure 1.**
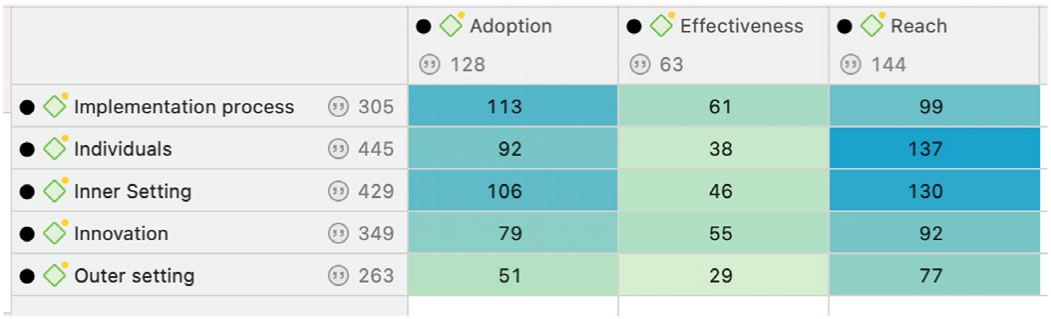
CFIR + RE-AIM co-occurrences in Atlas.Ti.

After initial coding was completed and priority RE-AIM outcomes identified, the team conducted a second round of coding focused on co-occurrences between the five CFIR domains and the three priority RE-AIM outcome. Figure 1, a screenshot from Atlas.Ti, shows the total number of co-occurrences between the three priority RE-AIM outcome codes and five CFIR domain codes. SM, JF, and EL were assigned quotations coded with both a priority RE-AIM outcome and the CFIR domains.

SM, JF, and EL re-coded quotations coded under their assigned co-occurrences with another set of codes designed to identify quotations of particular importance to this formative D&I analysis. SM, JF, and EL wrote memos to document priority CFIR constructs that appeared within quotations. CFIR constructs are smaller units of analysis that address more specific barriers and facilitators and exist within the five CFIR domains. This second round of coding and memoing assisted in identifying interrelations between the three key RE-AIM outcomes and main CFIR barriers and facilitators.

SM and HL then used the CFIR + ERIC Matching Tool to identify which expert-recommended implementation strategies would best potentially overcome likely TeamMAPPS implementation barriers identified by participants under “adoption” and “reach” [32]. We provide full results from our CFIR-ERIC Matching Tool analysis as supplementary material, including a document describing how we mapped constructs from the original 2009 CFIR to the updated 2022 CFIR (see, S1, S2, and S3) [28,47]. Matching tool outputs were combined with implementers’ stated needs in interviews and strategies recommended by the TeamMAPPS leadership team at UTMB (particularly KW, SM, and EL) to suggest implementation strategies.

## Findings

We report findings by RE-AIM outcomes that emerged as most critical: “adoption,” “reach,” and “effectiveness.” Within each, we report how participants spoke about key CFIR barriers and facilitators in descending order from most to least important based on statements in interviews. CFIR is organized into five high-level “domains” that refer to factors that can influence intervention implementation. Each CFIR domain in turn contains constructs, concepts that outline more specific categories of barriers and facilitators. Table 4 shows key RE-AIM outcomes along with CFIR domains and constructs. Table 5 shows select participant quotations within the “adoption,” “reach,” and “effectiveness” RE-AIM outcomes, organized by CFIR domains.

**Table 4.** Important CFIR constructs, barriers, and facilitators for each priority RE-AIM outcome

Key RE-AIM outcomes ordered by priority, with CFIR domains and constructs	Innovation	Outer Setting	Inner Setting	Individuals	Implementation Process
**Adoption**	Evidence-base(f) Adaptability(f) Source(f) Relative Advantage (f) Design (f)	External pressure (b/f)* Financing (b)* Local attitudes (b)* Local conditions (b)*	Work infrastructure (b/f) Culture (b/f)* Relational connections (f) Learning-centeredness (f) Deliverer-centeredness (f)	Implementation facilitators (b/f)* Implementation leads (f) Opinion leaders (f) High-level leaders (f) Implementation team members (b/f)* Innovation recipients (b/f)	Adapting (f) Planning (b) Assessing context (b/f) Assessing needs (b/f) Reflecting and evaluating (b)*
**Reach**	Evidence base (f) Design (f) Adaptability (f) Trialability (f) Relative Advantage (b)* Complexity (b)*	Local attitudes (b)* Partnerships and connections (f) Financing (b)* External pressure (b/f)*	Relational connections (f) Compatibility (f) Incentive system (b)* Mission alignment (f) Available resources (b)*	Mid-level leaders (b) Implementation facilitators (f) Innovation recipients (b)*	Tailoring strategies (f) Engaging (b)* Adapting (f) Reflecting and evaluating (b)*
**Effectiveness**	Evidence base (f) Source (f)	Partnerships and connections (f) Local attitudes (b)*	Relational connections (f) Culture (b/f)* Recipient-centeredness (b/f)*	Innovation recipients (b/f)* Innovation deliverers (b/f)* High-level leaders (b/f)*	Assessing needs (b/f)* Innovation deliverers (b/f)* Innovation recipients (b/f)* Reflecting and evaluation (b/f)* Assessing context (f) Tailoring strategies (f)

^1^Constructs are elements within the five CFIR domains; each CFIR domain has its own sub-constructs (see [28]).

^2^Key constructs are presented in descending order of importance to TeamMAPPS implementation, based on our participants’ responses in interviews.

*Denotes critical barrier, based on participants’ responses in interviews.

(b) = barrier; (f) = facilitator. CFIR: Consolidated Framework for Implementation Research; RE-AIM: Reach, Effectiveness, Adoption, Implementation, Maintenance and Sustainability.

**Table 5.** Demonstrative participant quotations at the intersection of key RE-AIM outcomes and CFIR domains

	Innovation (CFIR Domain)	Outer Setting (CFIR Domain)	Inner Setting (CFIR Domain)	Individuals (CFIR Domain)	Implementation Process (CFIR Domain)
Adoption (RE-AIM Outcome)	“I would swap probably two [other Team Science training modules] for two of the TeamMAPPS modules, and we would do a similar sort of asynchronous prep, and then come together for a half-day workshop and facilitated discussions and exercises by local people. I do think your interface and your videos and all of that would be much more attractive than what we currently use and much more informative as well. Much more science based as well. So, there’s a lot of advantages of your TeamMAPPS.” **(P014, Implementer)**	“Both the NIH, with the CTSAs, and the NSF, with its “convergent research” approach, are… translational. That’s my current reading of what NIH and NSF are articulating in terms of how they want scientists and practitioners to collaborate and move forward and, you know, address big problems. So, I would say [TeamMAPPS is] primarily [for] professionals in those fields, but it could also be applicable pretty broadly. I’m thinking about research development professionals who sort of help guide some of these teams at their universities.” **(P002, Conceptualizer)**	“One thing to look at is the leadership. What did they do to promote all this? The second one is, it has to be the PIs, you know, the leaders of the grant or the contract of the CTSA. Do they embrace these things? I’ve been involved as consultant to several CTSAs…there’s vast differences.” **(P004, Conceptualizer)**	“It would be a resource for, you know, research team members in our CTSA to use to become educated about teamwork and about Team Science …One idea I have is that it would be something that would be probably required of all the people that we would consider to be trainees in our CTSA.” **(P007, Implementer)**	“I definitely wouldn’t just say “this thing is available to use”…some people will make it mandated.” **(P003, Conceptualizer)** “It’s going to have to be given in bits and pieces that are relevant at a time that the team is ready to accept it, and learn it, and apply it. Then the other strategy, which I have not yet thought through [is] that the most effective training is a team-based training, not an individual training. Individuals who get trained on how to work in teams don’t always apply that.” **(P010, Implementer)**
Reach (RE-AIM Outcome)	“You can get all of this training, step right into the lab with a PI who doesn’t listen, who has a real like “I’m running the show,” and we’re going to see it lost in the water. So, really if you wanted to get this to be impactful… you’d be altering social interaction dynamics with the whole [team]. Then the practice episodes were a chance for people to unlearn and relearn new ways of interacting. Because it’s one thing to say, “oh, I heard someone do this, and I can see that the text, and I can see the way that they’re facilitating or creating psych safety,” or whatever the construct is. It’s a whole other thing in practice.” **(P001, Conceptualizer)**	“NCATS recently let us know that they want us to start educating people on the idea of not “translational research,” but the idea of “translational science”…We’re going to be adding some additional [elements in our pilot awards]. They won’t really be requirements; they’ll more be like “extra credit.” Like you can get higher scoring if you do something related to NCATS’s “translational science.” So, if we sort that out, and that seems sufficient, and that seems appropriate, then maybe we’ll do the same thing with Team Science.” **(P021, Implementer)**	“We do have two Team Science grants. They’re only $10,000 to start to help in kind of establishing these groups. Last year we didn’t really have much that was affiliated with it, but this year we’re probably going to put in that. It’s only two. So, it would be two teams that would have to go through the TeamMAPPS training.” **(P006, Implementer)**	“A lot of people will read the definition of what TeamMAPPS is and be like, “oh, I already know how to do that,” but they don’t really know…This kind of relates to what I was saying earlier about making the implicit explicit, and that we all go through these issues, but we don’t really think about how to deal with them or realize that we should deal with them…Framing the training is going to be really important, if it’s not going to be just another e-mail that somebody ignores.” **(P003, Conceptualizer)**	“I’m a big fan of a hybrid approach…meeting in the classroom and online. It doesn’t necessarily mean *just* that. It means identifying those parts that are the basic steps, right? Your process, your procedures, your work tasks…You’re going to show the video, then you’re going to ask questions, and then they’re going to do a reinforcing exercise. Then you get to tell a story that is consistent with what you’ve just taught…You do it in such a way that it’s not killing your learners with boredom and sameness…That for me is how [TeamMAPPS] exists as a hybrid approach.” **(P015, Designer)**
Effectiveness (RE-AIM Outcome)	“I think there’s potential there. I think that the framework is – I don’t want to say “universal” – but it’s basal enough that it can be applied to multiple disciplines that people can connect with it and engage with it, even bringing in their own personal and professional experiences. So, I think the potential is there, but I think that somebody has to feel like it has meaning for them.” **(P013, Implementer)**	“If you’re trying to improve teamwork on science teams, you would want to look at more proximal or upstream outcomes related to the to improved teamwork…to improve teamwork on science teams and reduce conflict. Other measures of improved communication, attitudes, and maybe, I think, the efficiency that the time that it takes to do research should go down with better communication and teamwork. Maybe job satisfaction, those kinds of things too.” **(P007, Implementer)**	“Teams have some basic principles that they adhere to that makes them a stronger team, and people have been studying that. That is evidence: “If you do this on your team, if you have these types of people, if you have diversity, if you have that, [but] how is that something that a scientist can take and be like, ‘okay, but I have this team right here. What do I do?’ That’s where…I think TeamMAPPS is: “you need to be able to do this, and this, and this, and this” – and I think that’s going to be very beneficial.” **(P006, Implementer)**	“Having high quality facilitators is important …You probably don’t have experts at all these places, including my own, of people who have read widely on the topic, and know some of the frameworks, and know the literature, and that sort of thing. It’s difficult to get someone at one of your user institutions to get up to speed on all of the Science of Team Science, but there certainly are people at the institutions you’d work with who could facilitate discussions and lead people through activities where…they would appreciate the value of Team Science.” **(P014, Implementer)**	“Something we might do for staff and or faculty to make it more meaningful than just, like, completion and testing against some questions, is we might give them some guiding prompts, right? Like “write a one-page reflection on how you’re going to change things, submit it to us, and then we’ll give you a locally recognized certificate.” **(P021, Implementer)**

CFIR: Consolidated Framework for Implementation Research; CTSA: Clinical and Translational Science Award; RE-AIM: Reach, Effectiveness, Adoption, Implementation, Maintenance and Sustainability; NIH: National Institutes of Health; NSF: National Science Foundation; PI: Principal Investigator; TeamMAPPS: Team Methods to Advance Processes and Performance in Science.

Conceptualizers tended to discuss high-level issues related to TeamMAPPS concepts, delivery, and evaluation. They generally emphasized issues that different institutions might face and offered recommendations to enhance implementation. Designers emphasized their decisions about how they built the online modules, often with reference to theories of adult learning and online or hybrid curriculum design. Several described the delivery strategies they thought were best for adult learners, including a preference for group activities rather than lectures if delivered in a hybrid model. Implementers tended to focus on strategies they planned to use at their institution, or reflected on what might work best.

In the following subsections, CFIR domains are presented in “quotation marks” and CFIR constructs are presented in *italics* to distinguish domains from constructs. Constructs are smaller units of analysis that exist within higher-order CFIR domains.

### Adoption barriers and facilitators

RE-AIM defines adoption as “the proportion and representativeness of settings…that adopt a given policy or program” [29,46]. In this formative pre-implementation D&I study, “adoption” emerged as a key TeamMAPPS implementation outcome. We focused our analysis of adoption on how participants framed likely barriers and facilitators at their institutions along with general anticipated adoption issues.

Within “innovation,” participants saw great benefit to adopting TeamMAPPS and described strategies to enable adoption that they planned to use. This enthusiasm reflected the characteristics of participants as individuals involved in the development, design, and implementation planning for TeamMAPPS. Participants consistently expressed that there is not a similar available intervention for science teams and spoke to the gap that TeamMAPPS can fill, emphasizing its *relative advantage*.

Among the most critical CFIR domains under adoption were “implementation process” and “inner setting.” Within “implementation process,” the *adaptability* of TeamMAPPS was seen as a major facilitator. The ability of TeamMAPPS to be delivered as a whole-team intervention, to be taken independently by individual scientists, or as part of a course was seen as a benefit. The adaptability of TeamMAPPS also appeared to influence *planning* processes for TeamMAPPS, particularly regarding institutional contingencies that would structure implementation. For example, CTSAs issue pilot awards, and some participants mentioned plans to incorporate TeamMAPPS into award requirements. Others described plans to incorporate TeamMAPPS into courses for trainees, as part of IPE, or as part of Team Science trainings and offerings. Participants also discussed barriers and facilitators related to *assessing context* and *assessing needs* of universities and CTSAs operating within the constraints and expectations of the health sciences, NIH, NCATS, and translational science. Several also spoke about the process of individuals *reflecting on and evaluating* their experiences implementing or receiving TeamMAPPS. While TeamMAPPS was perceived to be evidence-based and rooted in SciTS principles, participants noted that it would ideally be supported by additional evidence of effectiveness as implementation proceeded, through overall and site-specific evaluation.

Under the “inner setting” CFIR domain, participants discussed the importance of *work infrastructure* and *culture* at implementing institutions, particularly regarding systems of support for Team Science trainings. This was connected to the *culture* of institutions regarding Team Science. Some participants noted relatively limited support for trainings, while others described how their institutions valued and invested in Team Science. This fed into discussions about the *relational connections* that TeamMAPPS implementers could leverage at their institutions to disseminate it. TeamMAPPS’s flexibility was perceived as contributing to its *learner-centeredness* and *deliverer-centeredness*.

The second most discussed set of CFIR domains affecting adoption were “individuals” and “innovation.” Within “individuals,” the roles of *implementation facilitators*, *leads*, and *team members*, as well as *opinion leaders* and *high-level organizational leaders* were all framed as critical to adoption. An implementer’s ability to access and learn from the experts who developed TeamMAPPS was seen as crucial to facilitating widespread and effective adoption. However, the success of TeamMAPPS was seen to also rely on support from local leadership. Further, the flexibility of TeamMAPPS delivery was seen as a benefit to implementers and *innovation recipients*. Options discussed included using TeamMAPPS with students in classes, in trainee mentorship plans, with individual scientists, or with whole teams. Regarding “innovation,” TeamMAPPS was seen as being *evidence-based.* Implementers held the creators (*source*) of TeamMAPPS in high regard and believed that TeamMAPPS was highly *adaptable,* owing to the *design* of the modules and implementation support materials. These factors were seen supporting the adoption of TeamMAPPS. Adaptability also contributed to the *relative advantage* of TeamMAPPS compared to other available trainings.

Participants also spoke about factors that may influence TeamMAPPS adoption related to “outer setting.” That TeamMAPPS can be used to fulfill requirements for IPE and trainee activities led participants to frame TeamMAPPS as responsive to *external pressures* put upon implementing organizations by funders such as NIH, accreditors, and similar entities. Support from external entities, such as NIH, that *finance* Team Science and thereby foster *local attitudes and conditions* were also seen as critical to facilitating adoption.

### Reach barriers and facilitators

Reach was identified as a key implementation outcome. Reach refers to the number, proportion, and representativeness of potential end-users and their reasoning for/against implementation [40,46]. Generally, interview participants focused on the kind of end-users to target and how to provide compelling rationales to encourage participation. Much discussion centered around trade-offs of focusing on specific categories of learners and how to best balance benefits and costs. The hierarchy of academic research was discussed across nearly all domains and constructs. Relative positions of power were reported to influence whether an end-user may be interested in or capable of participating in the training and integrating Team Science principles into their work. The most emphasized CFIR domains related to reach were “individuals” and “inner setting.”

When discussing “individuals,” participants focused on the importance of *mid-level leaders* and *innovation recipients*. Mid-level leaders were felt to be critically important to maximize reach, due to their influence – particularly principal investigators, because they are often members of multiple teams and can influence junior researchers. Participants weighed benefits and costs of targeting specific individuals or groups for implementation. For example, it was felt that it might be beneficial to implement TeamMAPPS with entire teams, but drawbacks included lack of time and potential difficulties in a group activity with a dysfunctional team. The benefits and drawbacks of targeting junior rather than senior researchers were also considered. On one hand, junior investigators may have more time and may be more open to changing their teamwork style. On the other, senior investigators have more influence, but also more constraints on their time and potentially more solidified teamwork styles.

The most discussed constructs related to “inner setting” were *compatibility* and *available resources*. Compatibility was explored in terms of logistics related to participation. Like discussions of adoption, time was considered a major factor influencing reach. Investigators who need or want to participate may not be able to because of competing demands. However, participants identified many potential inner setting resources that might facilitate reach. Existing educational programs, pilot funding, and research training programs were considered promising vehicles.

“Implementation process” and “innovation” were also discussed. For implementation process, *tailoring strategies*, *adapting*, and *engaging* constructs often co-occurred. Tailoring mode of delivery was suggested as method of making TeamMAPPS more appealing. Tailoring by problems that a particular team is addressing, team maturity (i.e., newly formed vs. longstanding), stage of the research pathway (e.g., bench vs. animal vs. human trials), and individuals’ roles in the team were suggested as potentially helpful. Various methods of adapting delivery were also suggested to increase reach, such as offering hybrid delivery, delivering specific modules, or using learning contracts. Within the “innovation” domain, the *evidence base* of TeamMAPPS was a major perceived benefit to facilitate reach.

“Outer setting” was also discussed, though less than other domains. *Financing* along with *partnerships and connections* were two domains of note. The CTSA network was often discussed as an important part of the implementation context. Specifically, CTSAs were thought to facilitate Team Science training through incentivizing trainees to take them.

### Effectiveness barriers and facilitators

Effectiveness emerged as a key RE-AIM implementation outcome. RE-AIM defines effectiveness as “the impact of an intervention on important outcomes, including potential negative effects, quality of life, and economic outcomes” [29]. Because TeamMAPPS was not yet being implemented when we conducted our interviews, discussions about effectiveness were speculative and did not refer to observed effectiveness; rather, they spoke about the potential for effectiveness. Therefore, we focus on how participants defined effectiveness contextually and how it should be measured. Effectiveness was believed to be dependent on factors related to implementation fidelity – the quality of TeamMAPPS delivery – and the success of implementation efforts regarding adoption by institutions and reach to individual learners.

Many participants noted the importance of understanding the unique learning styles and career needs of individuals and the quality of the training experience. Ideally, a well-designed intervention that has evidentiary rigor and can be tailored to specific team needs could be quite effective for enculturating a collaborative group into a high functioning research team. While participants highlighted the effectiveness and utility of TeamMAPPS in these regards, they also discussed the need for quality assurance analyses to assess the fidelity of training and “transfer of training” across different intervention recipients. This reflected participants’ general desire for consistent training and application of Team Science principles. One interviewee articulated it as a “hybrid-model” whereby core elements would be standardized, while salient, team-specific guidance could also be incorporated to maximize effectiveness. Participants also suggested that there be mechanisms and metrics to demonstrate effectiveness, ranging from traditional measures of output (i.e., publications) to pre-/post-assessments of Team Science knowledge, skills, competencies provided by the program. Some of these, such as pre-/post- tests, are built into the TeamMAPPS modules. Others, such as measuring long-term outcomes, could become goals of this D&I study or local evaluations.

The main CFIR domains that emerged for the effectiveness outcome included “implementation process” and “innovation.” Regarding implementation process, participants noted their beliefs that the effectiveness of TeamMAPPS will hinge on careful reflection regarding *innovation recipients* and *innovation deliverers*. Specific concerns included pre-/post- assessments, assessing current team environment, making material meaningful and relevant, having authentic trainers, and measuring outcomes. This aligns with other domains including assessing context and tailoring strategies to optimize impact and ensure implementation fidelity. Regarding “innovation,” one of the most notable constructs was the *evidence base* used to develop TeamMAPPS, which helped establish its credibility. The intervention’s effectiveness was framed as likely being positively impacted by the *innovation source*, or the fact that Team Science leaders created the trainings using a robust evidence base.

Secondary CFIR domains that participants discussed in relation to effectiveness included “inner setting,” “individuals,” and “outer setting.” Within inner setting, the domains *relational connections*, *culture*, and *recipient centeredness* were prominent. Using TeamMAPPS to foster relational connections conducive to authentic interactions, understanding other team members’ roles, and reciprocity were deemed important implementing TeamMAPPS with fidelity to maximize effectiveness. *Cultural contexts* such as clarifying norms and creating aligned guiding principles focused on matters related to interpersonal relationships and navigating different team cultures. A focus on training recipients can foster the aforementioned issues to create a personalized experience that meets individual and team needs. In discussions related to “individuals,” *innovation recipients* and *innovation deliverers* were the two primary constructs. Participants often discussed these constructs concordantly, regarding the ability of TeamMAPPS to facilitate effective knowledge transfer, change team norms, meet user needs, and incentivize participation. Regarding “outer setting,” the primary domains that emerged were *partnerships and connections* coupled with *local attitudes*. Participants noted that leveraging CTSA hubs and networks and the support of high-level leaders may increase participation, buy-in, and overall implementation effectiveness. Supportive local context emerged as critically important.

### Potential TeamMAPPS implementation strategies

This section describes recommended implementation strategies. These were created based on findings from our interviews, recommendations from the TeamMAPPS leadership team, and findings from the CFIR + ERIC Matching Tool based on key CFIR barriers presented in Table 4 above. We exclude strategies related to “effectiveness,” as they would have been too speculative during pre-implementation. Table 6 shows potential implementation strategies, organized by the “reach” and “adoption” RE-AIM outcomes.

**Table 6.** Potential implementation strategies to enhance the adoption and reach of TeamMAPPS

	Recommended by TeamMAPPS leadership team	Recommended by TeamMAPPS D&I study participants	Recommended by CFIR + ERIC Matching Tool (top 15 strategies for Reach and Adoption)
**Reach + Adoption**	● Asynchronous administration followed by facilitated session ● Facilitated whole-team administration ● Host Train-the-Trainers for TeamMAPPS facilitators ● Offer TeamMAPPS as part of Team Science Core consultations ● TeamMAPPS certification badging ● Inclusion in required Responsible Conduct of Research (RCR) courses ● Creation of incentive systems to go through TeamMAPPS ● Maximizing the flexibility of the online platform ● Leveraging experience with TeamSTEPPS implementation to use in TeamMAPPS implementation	● Use in classes for graduate students and postdoctoral fellows ● Use in required IPE activities for school accreditation ● Incorporation into training section of grants (e.g., KL2s, T32) ● Inclusion of TeamMAPPS in team onboarding materials (e.g., lab orientation packets, new faculty on-boarding) ● Taking a combined “carrot” (e.g., incentives) and “stick” (e.g., requiring) approach to encourage uptake ● Leveraging experience with TeamSTEPPS implementation to use in TeamMAPPS implementation	● Capture and share local knowledge ● Assess for readiness and identify barriers and facilitators ● Conduct local needs assessment ● Conduct local consensus discussions ● Obtain and use patients/consumers and family feedback* ● Identify and prepare champions ● Involve patients/consumers and family members* ● Build a coalition ● Tailor strategies
**Reach Only:**	● Make TeamMAPPS available to individual investigators to go through on their own	● Emphasizing the TeamMAPPS evidence base to potential participants	● Alter incentive/allowance structures ● Access new funding ● Conduct educational meetings ● Inform local opinion leaders ● Conduct cyclical small tests of change ● Promote adaptability
**Adoption Only:**	● Forming a community of practice	● Inclusion in CTSA pilot award requirements ● Once-per-month TeamMAPPS sessions led by Team Science Cores ● Local facilitators trained to be experts in TeamMAPPS	● Develop and implement tools for quality monitoring ● Audit and provide feedback ● Create a learning collaborative ● Facilitation ● Use advisory boards and workgroups ● Involve executive boards

CFIR: Consolidated Framework for Implementation Research; CTSA: Clinical and Translational Science Award; ERIC: Expert Recommendations for Implementing Change; TeamMAPPS: Team Methods to Advance Processes and Performance in Science; TeamSTEPPS: Team Strategies and Tools to Enhance Performance and Patient Safety.

* The language of “patients” included in the CFIR + ERIC Matching Tool outputs reflects the tool’s original development for clinical interventions.

The first column displays implementation strategies recommended by the TeamMAPPS leadership team, of which this D&I study team is part. TeamMAPPS was designed with implementation strategies in mind, and there is an existing set of materials developed based on expert opinion available upon request that will be regularly updated. The second column shows recommendations based on participants’ interviews and the analysis above. The third column shows recommendations from the CFIR + ERIC Matching Tool, most of which map onto similar strategies in the first two columns. Notably, the CFIR + ERIC Matching Tool recommended many strategies that involve providing incentive structures for scientists to go through TeamMAPPS and shorting up local support. The creation of local assessment packages and tailored implementation strategies are also key recommendations.

These results provide diverse strategies to implement TeamMAPPS that could enhance reach and adoption. They were developed based on the triangulation of multiple different perspectives. CTSAs and other institutions seeking to implement TeamMAPPS should consider what strategies would work best for them based upon available resources and local needs, using our recommendations as guidelines.

## Discussion

Our findings are of pre-implementation data from early adopters designed to guide future TeamMAPPS implementation and to inform D&I-focused evaluation of Team Science interventions [30]. Review of Table 6 reveals several broad categories of implementation strategies for TeamMAPPS that may be generalizable beyond TeamMAPPS and applicable to other Team Science interventions. These involve the use of tailored implementation strategies built for specific institution; advocacy and support for Team Science; creating specific program delivery recommendations; ensuring flexibility of implementation; conducting local needs assessments; providing orientations for trainings; allowing participants to give feedback; and fostering support for Team Science trainings by building local committees of leadership and others to support implementation.

This study has several limitations. Data were gathered during pre-implementation, and participants mainly consisted of CTSA-affiliated Team Science professionals. They were aware of the potential value of Team Science trainings and familiar with the lexicons and values of SciTS and translational science. This was also a potential strength because this group held deep knowledge about barriers and facilitators in past efforts to implement trainings. One of our foci for the next phase of this study will be diversifying participant perspectives because we recognize that there will be different barriers to implementing TeamMAPPS at organizations without robust Team Science offerings. Our participants were aware of these barriers and discussed the relative marginalization of Team Science. Speaking to individuals about TeamMAPPS who might be skeptical of Team Science will allow us to develop implementation strategies for such people and organizations.

Based on findings presented here and our ongoing D&I study, our team is refining existing implementation materials to include packages for institutions that adopt particular strategies. We are especially drawing on recommendations in Table 6, combining ERIC strategies with those recommended by study participants and the TeamMAPPS team. We will workshop and discuss materials with individuals and institutions who use them as part of their TeamMAPPS implementations. This will be executed as part of Tasks four and five of our Implementation Mapping process during next steps of the D&I study, to facilitate the validation of “implementation bundles,” to be developed in consultation with implementers.

We conclude by noting what our participants spoke about as the greatest implementation barriers, which were scientists’ time to undergo trainings, attitudes about team trainings, and lacking institutional Team Science resources. As this D&I study develops, we aim to explore how the nascent TeamMAPPS community of practice has overcome similar barriers in past initiatives and this one. Ethnography and qualitative research are powerful tools for D&I Science, particularly in dense organizational ecologies like the CTSA network. Crucially, this style of inquiry exists in a liminal space enabling critical analysis as a participant-observer [48–50]. A strength of our study is that we are embedded in the CTSA network and part of the team implementing TeamMAPPS. We can thus work transversally across entities involved in this effort to understand and overcome barriers. As the use of evidence-based approaches to improve scientific teams becomes increasingly important, understanding how to best implement team trainings will be essential.

## Supporting information

Molldrem et al. supplementary material 1 Molldrem et al. supplementary material

Molldrem et al. supplementary material 2 Molldrem et al. supplementary material

Molldrem et al. supplementary material 3 Molldrem et al. supplementary material

## Supplementary material

The supplementary material for this article can be found at https://doi.org/10.1017/cts.2025.22.
