# Supplementary material for: Implementing TeamMAPPS: Formative qualitative findings from the dissemination and implementation study of a new evidence-based team science intervention
Source: J Clin Transl Sci. 2025 Feb 10;9(1):e68. doi: 10.1017/cts.2025.22 (PMC11975785; doi:10.1017/cts.2025.22)
Supplement: Molldrem et al. supplementary material 1 — Molldrem et al. supplementary material [file S2059866125000226sup001.docx]

This document describes how we mapped domains from the 2009 CFIR to the updated 2022 CFIR in entering barriers into the CFIR-ERIC Matching Tool for “adoption” and “reach” outcomes.

2009 CFIR domains and construct are underlined (see, [47]). 2022 Updated CFIR domains and constructs are in **bold** (see, [28]). Where language co-occurs fully between the 2009 and 2022 CFIR definitions, items are **bold underlined**.

**Key CFIR Barriers Identified for “Adoption”**:

- Intervention Characteristics/**Innovation**
  - No barriers identified
- **Outer setting**
  - Patient needs and resources = **Local attitudes** and **Local conditions**
  - External policy and incentives = **Financing** and **External Pressure**
- **Inner setting**
  - **Culture** = **Culture**
- Characteristics of Individuals/**Individuals**
  - Self-efficacy = **Implementation facilitators** and **Implementation team members**
- Process/**Implementation process**
  - **Reflecting and evaluating** = **Reflecting and evaluating**

**Key CFIR Barriers Identified for “Reach”**:

- Intervention Characteristics/**Innovation**
  - **Relative Advantage** = **Relative Advantage**
  - **Complexity** = **Complexity**
- **Outer setting**
  - External policies and incentives = **Financing** and **External Pressure**
  - Patient needs and resources = **Local attitudes**
- **Inner setting**:
  - Organizational incentives and rewards = **Incentive system**
  - **Available resources** = **Available resources**
- Characteristics of Individuals/**Individuals**
  - Knowledge and beliefs about the intervention; Individual stage of change = **Innovation recipients**
- Process/**Implementation process**
  - **Reflecting and evaluating** = **Reflecting and evaluating**
  - Patients and customers = **Engaging**
